# *CRP* genotype and haplotype associations with serum C-reactive protein level and DAS28 in untreated early rheumatoid arthritis patients

**DOI:** 10.1186/s13075-014-0475-3

**Published:** 2014-10-31

**Authors:** Christian Gytz Ammitzbøll, Rudi Steffensen, Martin Bøgsted, Kim Hørslev-Petersen, Merete L Hetland, Peter Junker, Julia S Johansen, Jan Pødenphant, Mikkel Østergaard, Torkell Ellingsen, Kristian Stengaard-Pedersen

**Affiliations:** Department of Rheumatology, Aarhus University Hospital, Nørrebrogade 44, 8000 Aarhus C, Denmark; Department of Medicine, Randers Regional Hospital, Skovlyvej 1, 8930 Randers, Denmark; Department of Clinical Immunology, Aalborg University Hospital, Urbansgade 32, 9000 Aalborg, Denmark; Department of Haematology, Aalborg University Hospital, Mølleparkvej 4, 9000 Aalborg, Denmark; Department of Mathematical Sciences, Aalborg University, Fredrik Bajers Vej 7G, 9220 Aalborg, Denmark; King Christian 10th Hospital for Rheumatic Diseases, Toldbodgade 3, 6300 Gråsten, Denmark; South Jutland Hospital, Institute of Regional Health Services Research, University of Southern Denmark, Winsløwparken 19, Odense M, Denmark; Copenhagen Center for Arthritis Research, Glostrup Hospital, Glostrup, Nordre Ringvej 57, 2600 Copenhagen, Denmark; Department of Clinical Medicine, Faculty of Health Sciences, University of Copenhagen, Blegdamsvej 3B, 2200 Copenhagen, Denmark; Department of Rheumatology C, Odense University Hospital, Sdr. Boulevard 29, 5000 Odense C, Denmark; Department of Medicine and Oncology, Herlev Hospital, Herlev Ringvej 75, 2730 Herlev, Denmark; Faculty of Health Sciences, University of Copenhagen, Blegdamsvej 3B, Copenhagen, Denmark; Copenhagen University at Gentofte, Niels Andersens Vej 65, 2900 Hellerup, Denmark; Department of Medicine, Silkeborg Regional Hospital, Falkevej 3, 8600 Silkeborg, Denmark

## Abstract

**Introduction:**

Single-nucleotide polymorphisms (SNPs) in the *CRP* gene are implicated in the regulation of the constitutional C-reactive protein (CRP) expression and its response to proinflammatory stimuli. Previous reports suggest that these effects may have an impact on clinical decision-making tools based on CRP, such as the Disease Activity Score in 28 joints (DAS28). We aimed to investigate the possible association between seven *CRP* SNPs, their haplotypes and the serum levels of CRP, as well as DAS28 scores, in two cohorts of untreated active early rheumatoid arthritis (RA) patients followed during their initial treatment.

**Methods:**

Overall, 315 patients with RA from two randomized controlled trials (the CIMESTRA and OPERA trials) who were naïve to disease-modifying antirheumatic drugs and steroids with disease durations less than 6 months were included. Seven *CRP* SNPs were investigated: rs11265257, rs1130864, rs1205, rs1800947, rs2808632, rs3093077 and rs876538. The genotype and haplotype associations with CRP and DAS28 levels were evaluated using linear regression analysis adjusted for age, sex and treatment.

**Results:**

The minor allele of rs1205 C > T was associated with decreased CRP levels at baseline (*P* = 0.03), with the TT genotype having a 50% reduction in CRP from 16.7 to 8.4 mg/L (*P* = 0.005) compared to homozygosity of the major allele, but no association was observed at year 1 (*P* = 0.38). The common H2 haplotype, characterized by the T allele of rs1205, was associated with a 26% reduction in CRP at baseline (*P* = 0.043), although no effect was observed at year 1 (*P* = 0.466). No other SNP or haplotype was associated with CRP at baseline or at year 1 (*P* ≥0.09). We observed no associations between SNPs or haplotypes and DAS28 scores at baseline or at year 1 (*P* ≥0.10).

**Conclusion:**

*CRP* genotype and haplotype were only marginally associated with serum CRP levels and had no association with the DAS28 score. This study shows that DAS28, the core parameter for inflammatory activity in RA, can be used for clinical decision-making without adjustment for *CRP* gene variants.

**Trial registration:**

The OPERA study is registered at Clinicaltrials.gov (NCT00660647). The CIMESTRA study is not listed in a clinical trials registry, because patients were included between October 1999 and October 2002.

**Electronic supplementary material:**

The online version of this article (doi:10.1186/s13075-014-0475-3) contains supplementary material, which is available to authorized users.

## Introduction

C-reactive protein (CRP) was discovered in 1930 by Tillet and Francis [1]. They realized that serum from febrile patients formed a precipitate when mixed with a *Streptococcus pneumoniae* capsule component due to binding of CRP to phosphorylcholine, a major constituent of C-polysaccharide. CRP is a highly conserved protein belonging to the pentraxin family and is a key component of the acute-phase response to infection and inflammation. CRP is in widespread clinical use as a sensitive but nonspecific marker of inflammation; a normal CRP expression level is below 10 mg/L in healthy individuals [2]. CRP levels may increase up to 1,000-fold following an acute-phase stimulus, owing to increased hepatic transcription, mainly in response to the proinflammatory cytokine interleukin 6 [2,3]. Several factors, such as age, sex, smoking and body mass index, have been shown to influence basal serum CRP levels in the absence of inflammatory stimuli [4-6].

**Figure 1:**
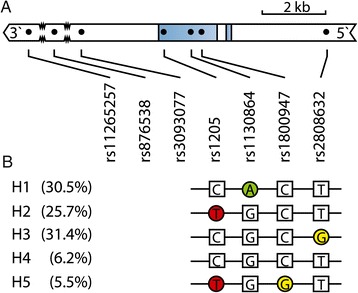
**Single-nucleotide polymorphisms and haplotypes in the** ***CRP*** **gene. (A)** Schematic representation of the *CRP* gene drawn to scale. Exons are represented as blue boxes. Dots represent the locations of the seven single-nucleotide polymorphisms investigated in this study. Zigzag lines indicate truncation of nucleotides. **(B)** Haplotypes and names are in agreement with those used in previous publications [16,17]. The nucleotides are stated according to the plus strand. The frequencies of haplotypes are given as percentages.

CRP is encoded by the *CRP* gene, which is located on chromosome 1q23. *CRP* consists of two exons spanning 2.3 kb (Figure 1). CRP levels are under genetic influence [7], and several single-nucleotide polymorphisms (SNPs) in the *CRP* gene and their haplotypes have been associated with basal CRP concentrations and the magnitude of the acute-phase rise in CRP levels in active inflammation [8-14]. Much of the work in this area has been instigated by the association found between elevated basal CRP levels and increased risk for cardiovascular disease [15].

Rhodes *et al*. reported a 3.5-fold change in expected serum CRP levels between carriers of two common *CRP* haplotypes in two cross-sectional cohorts of patients with chronic rheumatoid arthritis (RA) when the erythrocyte sedimentation rate was used to adjust for the varying degree of inflammatory disease activity [18]. Rhodes and colleagues speculated that genetic effects of this magnitude on CRP levels could result in suboptimal treatment due to the use of CRP-based (Disease Activity Score in 28 joints (DAS28)) treatment algorithms; for example, patients with low CRP production might receive inappropriate assurance or suboptimal treatment [16,18]. In contrast, Plant *et al*. recently published an analysis of a cohort of patients with chronic RA in whom they found no sign of an association between *CRP* variants and CRP, DAS28 or change in DAS28 after treatment with an anti–tumor necrosis factor inhibitor over a 6-month period [17]. In both studies, the researchers investigated patients with chronic RA who were being treated with a wide range of disease-modifying antirheumatic drugs (DMARDs), which could explain the diverging results. A cohort consisting of patients with early RA (ERA) who were DMARD- and steroid-naïve was unique to investigate, as they presented with the acute phase of chronic inflammation unaffected by the modifications of DMARD and steroid treatment.

Our aim was to investigate the importance of seven *CRP* SNPs in 315 patients with ERA who were DMARD- and steroid-naïve by determination of their genotype and haplotype associations with CRP and DAS28 score at baseline and after 1 year of treatment. The patients were derived from two previously conducted national randomized controlled trials.

## Methods

### Study participants

The study was based on two cohorts. Cohort 1 was derived from the OPERA study (ClinicalTrials.gov ID: NCT00660647) [19]. It was composed of 188 patients with ERA who were DMARD- and steroid-naïve were included in a randomized, double-blind, placebo-controlled trial of methotrexate, intra-articular (i.a.) glucocorticoids plus either adalimumab or placebo. Cohort 2 was composed of the patients in the CIMESTRA trial [20-22], which was a randomized, double-blind, placebo-controlled, investigator-initiated trial on 160 patients with ERA who were DMARD- and steroid-naïve and being treated with methotrexate and i.a. glucocorticoids plus either cyclosporine or placebo. DNA was available for all patients in cohort 1 and for 135 patients in cohort 2, so we had a total of 315 patients. The two cohorts had similar inclusion criteria. All patients fulfilled the 1987 American College of Rheumatology criteria for RA [23], had a disease duration of less than 6 months and were DMARD- and steroid-naïve. They had clinically active disease with a DAS28 score >3.2 (cohort 1) or at least two swollen joints (cohort 2). The treatment strategy of both studies was to achieve early and sustained synovitis suppression by aggressive use of i.a. glucocorticoids and DMARDs as a “treat-to-target” strategy [24]. The placebo groups of both studies were treated similarly (methotrexate and i.a. glucocorticoids), although the escalation rate of methotrexate and the type of glucocorticoid used (triamcinolone in OPERA and betamethasone in CIMESTRA) differed. Oral glucocorticoids were not allowed. There were only minor differences in patient characteristics between the two cohorts (Table 1). The median age of the included patients was 54 years; 66% were women; median disease duration was just under 3 months; and 70% were rheumatoid factor–positive. Serum CRP was measured at baseline for all patients and at year 1 for 170 patients in cohort 1 and 122 patients in cohort 2.

**Table 1:** **Patient characteristics of the two cohorts** ^**a**^

**Baseline**	**Cohort 1 (** ***N*** **=180)**		**Cohort 2 (** ***N*** **=135)**		***P*** **-value**
Age, yr	55	(27 to 78)	52	(26 to 71)	0.11
Female sex	66%		65%		0.91
Disease duration, days	84	(42 to 162)	98	(48 to 175)	<0.001
Rheumatoid factor–positive, %	72%		68%		0.54
Anti-CCP-positive, %	65%		60%		0.41
Tender joint count	11	(3 to 26)	9	(2 to 22)	0.07
Swollen joint count	8	(2 to 23)	8	(2 to 20)	0.41
Patient global assessment, mm	67	(13 to 98)	50	(10 to 91)	<0.001
CRP, mg/L	14	(1 to 132)	20	(2 to 105)	0.04
DAS28 score	5.6	(3.8 to 7.7)	5.3	(3.2 to 7.1)	0.02
HAQ	1.1	(0.1 to 2.5)	1.0	(0 to 2.3)	0.02
Year 1					
CRP, mg/L	2	(0.5 to 19)	7	(0.7 to 33)	<0.001
DAS28 score	2.0	(1.7 to 4.3)	2.0	(1.3 to 5.1)	0.13

^a^Values are medians with 5% to 95% percentile values in parentheses, unless otherwise stated. CCP, Cyclic citrullinated peptide; DAS28, Disease Activity Score in 28 joints; HAQ, Health Assessment Questionnaire. Fisher’s exact test and the Mann–Whitney rank-sum test were used when appropriate.

### Ethics

All patients gave their written informed consent, and ethical approval was obtained from the local ethics committee (De Videnskabsetiske Komitéer for Region Midtjylland, M-20110150).

### Single-nucleotide polymorphism selection and genotyping

The seven *CRP* SNPs studied (rs11265257, rs1130864, rs1205, rs1800947, rs2808632, rs3093077 and rs876538) were selected on the basis of previous publications in rheumatic diseases [16,18,25] (Figure 1). The nucleotides are stated according to the plus strand. Genotyping was analyzed using the TaqMan OpenArray system from Applied Biosystems (Foster City, CA, USA). We typed one SNP with a custom-designed genotyping assay and six SNPs with a predesigned assay from Applied Biosystems (see Additional file 1: Table S1 for assay information). We have previously described this method in more detail [26]. OpenArray plates were manufactured by Applied Biosystems. A nontemplate control was introduced within each set of assays. TaqMan OpenArray Master Mix was used according to the manufacturer’s protocol. Samples were loaded into OpenArray plates using the OpenArray NT Autoloader and cycled using the GeneAmp PCR System 9700 thermal cycler with PCR conditions set according to the manufacturer’s protocol. The arrays were read using the OpenArray NT Imager, and the allele calls and scatterplots were generated with the genotyping software associated with the OpenArray system.

### C-reactive protein measurements

CRP levels (range, 8 to 160 mg/L) in serum were measured using QuikRead go CRP (Orion Diagnostica, Espoo, Finland) [27] in cohort 1, and CRP values below 10 mg/L were reanalyzed with a high-sensitivity CRP assay to improve the sensitivity. CRP was measured using standard laboratory methods in each of the participating centers in cohort 2 [28].

### Statistical analysis

CRP concentrations in serum were log-normally distributed and, therefore, log-transformed before analysis. The genotype distribution was tested for deviation from Hardy-Weinberg equilibrium using Haploview software [29], which was also used for population-based haplotype analysis. Analysis of variance based on multiple linear regression models was used to investigate the association between CRP and genotypes with age, sex and treatment as covariates. The genotype was modeled as an additive allelic effect. The two cohorts were combined in the analysis of the genotype and haplotype effects.

Tests of haplotype association with protein levels were performed by using haplo.stats in the R statistical software package, version 1.6.3 [30]. For all analyses, we assumed additive haplotype effects and Gaussian distribution of traits and phenotypes. First, the haplo.score was used to study associations between the combined haplotypes and CRP levels by calculating global *P*-values. Next, the haplo.glm function was used to account for ambiguity of haplotype estimation and multiple comparisons so that we could estimate the effect of each haplotype compared to the most frequent haplotype (H3) by linear regression analysis. Age, sex and treatment were added as covariates in all haplotype analyses. In contrast to assigning the most likely haplotype phase resolution to each sample, the haplo.glm function estimates a generalized linear model by incorporating the haplotype phase uncertainty by inferring a probability matrix of haplotype likelihoods for each individual by use of the expectation-maximization haplotype inference algorithm.

Results with *P*-values below 0.05 were considered significant, and 95% confidence intervals (CI) are used throughout. Data are presented as medians (5th to 95th percentile ranges), unless otherwise stated. Fisher’s exact test, the Mann–Whitney rank-sum test and Student’s *t*-test were used to test population differences. Stata 12.1 software (StataCorp, College Station, TX, USA) was used for analysis.

## Results

No difference was observed between the two cohorts concerning age, sex, anti–cyclic citrullinated peptide or immunoglobulin M rheumatoid factor positivity (Table 1). There were significant differences in serum CRP levels between the two cohorts, with cohort 1 having the lowest levels. At baseline, cohort 1 had a median CRP level of 14 mg/L compared to 20 mg/L in cohort 2 (*P* = 0.04); at year 1, the median CRP level in cohort 1 was 2 mg/L compared to 7 mg/L in cohort 2 (*P* <0.001). Seventeen percent of the CRP measurements were >10 mg/L in cohort 1 compared to 40% in cohort 2 at year 1.

There were no differences between the two cohorts in the genotype frequency of the seven SNPs (all *P* ≥0.70). The minor allele frequency values (cohort 1/cohort 2) were rs11265257 (0.39/0.40), rs1130864 (0.31/0.30), rs1205 (0.32/0.31), rs1800947 (0.06/0.06), rs2808632 (0.31/0.33), rs3093077 (0.05/0.04) and rs876538 (0.22/0.24). These SNPs were genotyped in 315 samples. The genotyping success rate was >99%, and no deviation from the Hardy-Weinberg equilibrium was observed (all *P* ≥0.163) (Additional file 2: Table S2).

**Figure 2:**
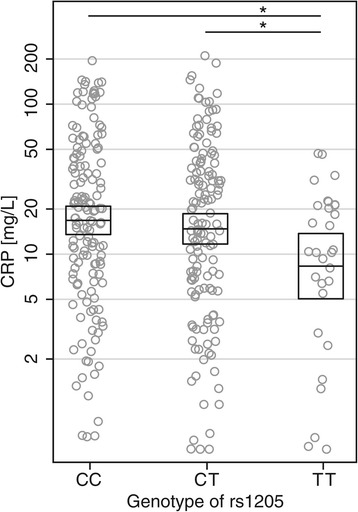
**Serum C-reactive protein concentrations divided by rs1205 genotype at baseline.** Serum C-reactive protein (CRP) concentrations of 313 patients with early rheumatoid arthritis at their time of diagnosis according to rs1205 genotype. Circles indicate individual serum CRP concentrations. The boxes indicate geometric means and 95% CIs calculated from linear regression analysis corrected for age, sex and treatment. *P <0.05.

Table 2 shows the association between SNPs and CRP as well as DAS28 levels, with sex, age and treatment as covariates. rs1205 (C > T) was associated with CRP levels at baseline in an allele dose-dependent way, with the minor T allele associated with lower values (*P* = 0.03). The regression analysis revealed a reduction to 50% CI (29 to 84) in baseline CRP levels for individuals homozygous for the minor T allele compared to the homozygosity of the major allele, and to 55% CI (33 to 94) compared to heterozygous for the minor T allele. Figure 2 gives a graphical presentation of the findings and demonstrates the large deviation in CRP in patients with ERA at diagnosis, with more than a 100-fold difference in serum CRP levels. No other SNPs were associated with baseline serum CRP levels, and none were significant at year 1, including rs1205 (all *P* ≥0.14). We found no genotype effect of the seven SNPs on DAS28 levels at either baseline or year 1 (all *P* ≥0.10) (Table 2).

**Table 2:** **Genotype effect on serum C-reactive protein level and Disease Activity Score in 28 joints at baseline and year 1** ^**a**^

	**Baseline CRP**		**Year 1 CRP**		**Baseline DAS28**		**Year 1 DAS28**	
**SNP**	**β**	***P*** **-value**	**β**	***P*** **-value**	**β**	***P*** **-value**	**β**	***P*** **-value**
rs11265257	−0.14	0.19	−0.01	0.91	−0.09	0.31	−0.06	0.49
rs1130864	0.15	0.21	0.03	0.78	0.16	0.10	0.14	0.12
rs1205	−0.26	0.03	−0.09	0.38	−0.08	0.39	−0.09	0.32
rs1800947	−0.34	0.14	−0.12	0.57	0.02	0.91	0.23	0.21
rs2808632	0.18	0.15	−0.04	0.73	−0.04	0.71	−0.08	0.40
rs3093077	−0.10	0.69	0.30	0.16	−0.26	0.21	−0.02	0.90
rs876538	0.15	0.28	−0.05	0.66	0.02	0.86	−0.02	0.87

^a^Linear regression analysis with age, sex and treatment as covariates. CRP, C-reactive protein; DAS28, Disease Activity Score in 28 joints; SNP, Single-nucleotide polymorphism.

Five haplotypes were constructed with frequencies between 6% and 32%, encompassing >99% of the cohort (Table 3 and Figure 1). These haplotypes are in agreement with the haplotypes previously published by Rhodes *et al*. [18] and Plant *et al*. [17]. We have named our haplotypes H1 to H5, similarly to Plant and colleagues and the authors of a recent review [16]. Rhodes and colleagues interchanged the haplotypes H4 and H5 [18]. “Haplo.score” revealed a significant global *P*-value of 0.04 between the combined haplotypes, and CRP levels at baseline and borderline were significant at year 1 (*P* = 0.06). There was no association between the combined haplotypes and DAS28 scores at either baseline (*P* = 0.10) or year 1 (*P* = 0.25), as judged by the global *P*-values. The results of the linear regression analysis (“haplo.glm”) are given in Table 3 as effects expressed in percentages compared to the reference haplotype H3. The H2 haplotype, characterized by the T allele of rs1205, was significantly associated with a reduction in CRP levels to 74% (55 to 99) (*P* = 0.043) at baseline, whereas no effect was observed at year 1. H5, which also includes the T allele of rs1205, was borderline significant at baseline with a reduction of 64% (40 to 103) (*P* = 0.07). No significant associations were seen between any of the other haplotypes and CRP levels at either baseline or year 1 (Table 3). No haplotype was associated with the DAS28 score at either baseline or year 1 (all *P* ≥0.23).

**Table 3:** ***CRP*** **haplotype build, frequency and effect on serum C-reactive protein and Disease Activity Score in 28 joints at baseline and year 1** ^**a**^

**Haplotype**	**Frequency**	**Baseline CRP, effect (CI)**	**Year 1 CRP, effect (CI)**	**Baseline DAS28, effect (CI)**	**Year 1 DAS28, effect (CI)**
H1 (CACT)	30.5%	96% (73 to 126)	99% (77 to 126)	102% (98 to 107)	105% (97 to 113)
H2 (TGCT)	25.7%	74% (55 to 99)	91% (70 to 118)	99% (94 to 103)	97% (89 to 106)
H3 (CGCG)	31.4%	100%	100%	100%	100%
H4 (CGCT)	6.2%	66% (41 to 107)	121% (80 to 184)	95% (88 to 103)	102% (89 to 116)
H5 (TGGT)	5.5%	64% (40 to 103)	86% (56 to 134)	101% (93 to 108)	110% (95 to 124)

Haplotype defining SNPs = (rs1205, rs1130864, rs1800947, rs2808632). The effects of each haplotype were given relative to the most frequent haplotype, which was used as reference. CRP, C-reactive protein; DAS28, Disease Activity Score in 28 joints; SNP, Single-nucleotide polymorphism.

## Discussion

To the best of our knowledge, this study is the first in which the associations between *CRP* SNPs, serum CRP levels and DAS28 scores have been investigated in a cohort of patients with inflammatory active early RA who were DMARD- and steroid-naïve. We found an association between rs1205 and serum CRP levels at baseline that translated into a reduction of 50% in CRP levels in patients homozygotic for the minor allele, but no such effects were observed at year 1. We found genetic variants in the *CRP* gene to be of questionable clinical significance in RA, as there was no association with DAS28 levels. Thus, we propose that DAS28 still can be used for clinical decision-making without adjustment for *CRP* gene variants.

The minor allele of rs1205 (C > T) was associated with decreased CRP levels at baseline. Homozygosity of the minor allele (TT genotype) was associated with a 50% reduction in CRP from 16.7 to 8.4 mg/L (Figure 1), which is in accordance with a similar 40% reduction described by Rhodes *et al*. [18]; however, Plant *et al*. did not find such an association (*P* = 0.17) [17]. Multiple studies with different patient populations have shown similar findings [12,13,25,31-33]. The absolute differences in two population-based studies of basal CRP were minute, with a difference in CRP of less than 1 mg/L between the CC and TT genotypes [12,32], but highly significant because of the high numbers of individuals included (*N* = 7,983 [12] and *N* = 3,107 [32]). Similar findings with a difference of about 30% following acute coronary ischemia have been described [31].

We did not find an association at year 1 between rs1205 and serum CRP levels, for which there are several possible explanations. CRP is the most commonly used biochemical variable to monitor disease activity, and, as both of the cohorts we included were derived from randomized controlled trials focused on aggressive, escalating treatment and tight disease control [19,20], this would bias the study by applying more aggressive treatment to the common variants of rs1205 (with higher CRP levels), thereby masking the genetic effect. A second explanation is that our study was underpowered to detect the 0.2 mg/L difference in CRP found at year 1 between the CC (4.6 mg/L) and TT (4.4 mg/L) genotypes.

We did not find a genotype or haplotype association with the DAS28 score at baseline, which is not surprising when one looks closely at the composition of the DAS28 equation [34]. The difference in CRP levels at baseline between the rs1205 CC (16.7 mg/L) and TT (8.4 mg/L) genotypes translates into a reduction in the DAS28 score of 0.23, which represents only 4% of the mean DAS28 score of 5.4 in the combined cohort. A power analysis revealed that a total of 1,343 patients should be included to detect such a change, with the estimated standard deviations of 1.12, a power of 0.80 and a significance level of 0.05. Plant *et al*. similarly observed no haplotype effects on DAS28 score or change in DAS28 score after 6 months of treatment with anti–tumor necrosis factor inhibitor therapy in 442 chronic RA patients [17]. Rhodes *et al*. reported a 3.5-fold change in expected CRP levels between the H1 and H5 (named H4 in the Rhodes article) haplotypes when the erythrocyte sedimentation rate was used to adjust for the varying degree of inflammatory disease activity, but their study lacked sufficient clinical data to report DAS28 scores [16]. They further speculated that genetic effects of this magnitude on CRP levels may result in suboptimal treatment because of the widespread use of CRP-based treatment algorithms (DAS28); for example, low CRP producers may receive inappropriate assurance or suboptimal treatment [18]. The absence of an association between geno- and haplotypes with DAS28 score in the present study accords with data previously reported by Plant *et al*. [17] and hence do not support concerns raised by Rhodes *et al*. [16,18] that genetically determined variability of CRP levels in serum may influence the assessment of disease activity in ERA or chronic RA.

The present study differs from two previously published studies regarding RA [17,18]. The patients had a median disease duration of 12 years in the Plant *et al*. study [17]. Although disease duration was not provided in the Rhodes *et al*. report [18], we assume a similar duration because their patients’ median age was 62 years. This is in contrast to our study, in which patients had treatment-naïve ERA at the time of inclusion (median disease duration of 3 months and median age of 54 years); that is, at the time of inclusion, our patients represented a fairly homogeneous cohort of untreated patients with ERA. It is unique to investigate untreated patients with ERA, as the rise in CRP associated with the appearance of RA is unaffected by treatment, rendering the analysis of the genetic effects on CRP levels more robust. Our study had fewer patients included than the two previous studies [17,18], but this is counterbalanced by the use of randomized controlled trial cohorts, short disease duration and the focus on untreated patients with ERA.

In this study, we focused on the hypothesis that genetically determined low serum levels of CRP may lead to underestimation of RA disease activity. Of note, however, high CRP production may also be harmful. First, CRP participates in the activation and regulation of all three pathways of the complement system [35], which is activated in RA [36]. Second, increased serum CRP concentration at baseline is an important predictor of subsequent death due to cardiovascular disease in patients with new-onset inflammatory polyarthritis, and it is independent of other indicators of disease severity [37]. Finally, several clinical studies have shown that high levels of CRP are associated with a more aggressive disease course and increased joint destruction [38].

## Conclusion

The *CRP* genotype and haplotype were only marginally associated with serum CRP levels and were not associated with DAS28 scores. Thus, this study shows that DAS28, which is the core parameter for inflammatory activity in RA, can be used for clinical decision-making without adjustment for *CRP* gene variants.

## Additional files

Additional file 1: Table S1. Assay information for the seven SNPs genotyped in 315 early RA patients. This table list the assay IDs, primers and probes used to analyze the seven *CRP* SNPs. Additional file 2: Table S2. Genotype data on the seven *CRP* SNPs investigated. This table lists the positions, major and minor alleles, Hardy-Weinberg equilibrium *P*-values, percentages successfully genotyped and minor allele frequencies in the combined cohort.

## Abbreviations

CRP: C-reactive protein
DMARD: Disease-modifying antirheumatic drug
ERA: Early rheumatoid arthritis
i.a.: Intra-articular
RA: Rheumatoid arthritis
SNP: Single-nucleotide polymorphism
