# Supplementary material for: CRP genotype and haplotype associations with serum C-reactive protein level and DAS28 in untreated early rheumatoid arthritis patients
Source: Arthritis Res Ther. 2014 Oct 31;16(5):475. doi: 10.1186/s13075-014-0475-3 (PMC4247621; doi:10.1186/s13075-014-0475-3)
Supplement: Additional file 1: Table S1. — Assay information for the seven SNPs genotyped in 315 early RA patients. This table list the assay IDs, primers and probes used to analyze the seven CRP SNPs. [file 13075_2014_475_MOESM1_ESM.pdf]

Supplementary table 1. Assay information for the 7 SNPs genotyped in 315 early RA patients

rs number	Assay id	Forward Primer	Reverse Primer	VIC Probe	FAM probe
**rs11265257**	C__11624376_10				
**rs1130864**	C___7479332_10				
**rs1205**	C___7479334_10				
**rs1800947**	C____177490_10				
**rs3093077**	C__32343424_10				
**rs876538**	C___7479344_10				
**rs2808632**	custom-designed assay	5'-TGGTACCAGGATAGTGGGATACTG	5'-TTCCAAGCTTTTCCACATCTTCCT	5'-TCTGCCCCTAGTTGGT	5'-TCTGCCCATAGTTGGT

Data on the forward and reverse primers regarding the not custom-designed assays are not available for commercial reasons.
