# Supplementary material for: CRP genotype and haplotype associations with serum C-reactive protein level and DAS28 in untreated early rheumatoid arthritis patients
Source: Arthritis Res Ther. 2014 Oct 31;16(5):475. doi: 10.1186/s13075-014-0475-3 (PMC4247621; doi:10.1186/s13075-014-0475-3)
Supplement: Additional file 2: Table S2. — Genotype data on the seven CRP SNPs investigated. This table lists the positions, major and minor alleles, Hardy-Weinberg equilibrium P-values, percentages successfully genotyped and minor allele frequencies in the combined cohort. [file 13075_2014_475_MOESM2_ESM.pdf]

Supplementary table 2. Genotype data on Hardy-Weinberg equilibrium and minor allele frequency on the combined cohort.

rs-nr	Position	Observed heterozygosity	Predicted heterozygosity	Hardy-Weinberg equilibrium p value	Successful genotyped %	Minor allele frequency	Major:Minor allele#
rs2808632	159687373	0.472	0.434	0.163	100	0.318	T:G
rs1800947	159683438	0.117	0.110	0.653	100	0.059	C:G
rs1130864	159683091	0.421	0.424	0.966	100	0.305	G:A
rs1205	159682233	0.446	0.434	0.751	99.4	0.318	C:T
rs3093077	159679636	0.098	0.094	0.921	99.7	0.049	A:C
rs876538	159675717	0.376	0.350	0.260	99.4	0.226	C:T
rs11265257	159668984	0.472	0.476	0.932	100	0.391	C:T

#Nucleotides are given according to the plus strand.
